# On the Temperature, Urea, Chloride of Sodium, and Urinary Water in Scarlet Fever, and on a Cycle in Disease and Health

**Published:** 1862-06

**Authors:** Sydney Ringer


MEDICAL JOURNAL.

VOL. V.] JUNE, 1862. [No. 6

### SELECTED.

### ON THE TEMPERATURE, UREA, CHLORIDE OF SODIUM, AND URINARY WATER IN SCARLET FEVER, AND ON A CYCLE IN DISEASE AND HEALTH.

BY SYDNEY RINGER, M. D.

*Communicated by Dr. Garrod, F. R. S.*

The observations were made on patients in the Hospital for Sick Children, under the care of Drs. West, Jenner and Hillier. Thirty cases are given. The temperature, taken several times during the day, is given in charts. The urea and chloride of sodium were estimated daily by Liebig's volumetric method. The observations extended over a variable time, in some cases till the forty-fifty day of the disease.

### I. *On the Temperature.*

1. This fell, in the great majority of cases, on either the fifth, tenth, or fifteenth day of the disease.

2. When the temperature remained high till the fifteenth or twentieth day, a fall of variable intensity occurred, usually on each of the preceding fifth days—namely, the fifth, tenth and fifthteenth. The temperature after each fall in some cases remained, during the subsequent five days, at the same point reached on the preceding fifth day; in other cases it rose again, reaching during the second or third five days a point as high as it did during the first five.

3. Each fall of the temperature is accompanied by an improvement in the state of the patient, which remains permanent when the temperature does not again rise.

4. Of seventeen cases that came early under notice, the average maximum temperature was a little above 103°.

5. Subsequent to the great fall experienced on the fifth, tenth, or fifteenth day, the temperature often remained rather too high over a variable time, in some cases for fifteen days. The degree of elevation varied, in some cases being between 100° to 101°, but more frequently between 99° and 100°. This elevation of the temperature also usually experienced a fall on each fifth day.

6. This subsequent elevation of the temperature, if of any persistence, was coincident with a continuation of the lesions produced by the scarlet fever, as sore-throat, &c. It sometimes preceded an attack of Bright's disease.

7. At a variable point after the scarlet fever, another elevation of the temperature occurred, due either to Bright's disease, endocarditis, tuberculosis, or chicken-pox; in two cases, the cause could not be ascertained.

8. The date of the second elevation varied; thus, counting from the commencement of the scarlet fever, in albuminuria the mean of six cases gave the twenty-second day; in two cases in which the elevation was probably due to endocarditis, the elevation began on the eighth day; in one case of chicken-pox, it commenced on the sixth day; in one case of tuberculosis, on the ninth.

9. The duration of the elevation due to the above causes varied from two to thirteen days.

10. This subsequent elevation of the temperature due to intercurrent disease always fell either on a fifth day from its own commencement or on a fifth day from the commencement of the scarlet fever.

11. Thus the temperature forms arcs of cycles, lasting in the majority of cases five days; this equality applies to the temperature of the scarlet fever, or of any subsequent intercurrent disease.

12. In severe cases the temperature remained at the same point throughout the day; in slighter cases it fell in the morning and rose during the day: this fall in the morning is one of the earliest signs of improvement.

13. The hour of the day at which the temperature reached its highest point varied greatly. It was most frequently at its highest at some time between two P. M. and eight P. M.

### II. *On the Urea.*

1. The urea appears to suffer no increase during the fever.

2. The amount of urea for many days after the decline of the fever is far below the amount normal to the patient.

3. From the above, the author thinks it probable that the kidney is affected from the commencement of the attack, and the elimination of the urea thus checked. In some of the cases the children were puffy about the face, without any blood or albumen occurring in the urine; this perhaps was caused by the retention of urea.

4. On the intercurrence of Bright's disease, the urea in some cases was greatly diminished; in other cases no diminution occurred.

### III. *On the Chlorides.*

1. The chlorides were never found absent in any of the cases analyzed.

2. Their amount was always much diminished during the fever days.

3. After the fall of the temperature the chlorides increased gradually.

4. In one case in which Bright's disease supervened the chlorides were estimated: they suffered very little diminution.

### IV. *On the Urinary Water.*

Often during the fever there is no diminution in the amount of urinary water; in some cases it is increased.

### V. *On the Albumen in the Urine.*

1. The albumen appears at two different periods: (*a*) during the fever days; (*b*) later, during the non-fever days. Out of 21 cases, it only appeared once during the fever days. Of 18 cases which were in the hospital for a considerable time, in 7 albumen appeared during the fever free days.

2. The time of its appearance varied from the ninth to the twenty-third day.

3. The duration of the albumen in the urine varied from three to forty-nine days.

4. There is no necessary connexion between the intensity of the inflammation (tested by the elevation of the temperature) and the duration of the albumen in the urine.

5. There is no necessary connexion between the intensity of the inflammation and the amount of albumen in the urine.

### VI. *On Blood in the Urine.*

1. There may be an elevation of the temperature, due probably to inflammation of the kidney, without any blood in the urine.

2. In no cases did blood appear without previous elevation of the temperature.

3. In some cases the blood continued long after the fall of the temperature, and thus probably after the decline of the inflammation.

### VII. *Relation between the Blood and Albumen in the Urine.*

1. A very large amount of albumen may occur in the urine without any blood.

2. Blood to a very large amount may occur in the urine with the slightest trace of albumen; and if the blood corpuscles be allowed to settle, the supernatant fluid may give no evidence of albumen.

These cases given were seldom dropsical; they, however, often looked puffy in the face. In some cases the second elevation of the temperature due to Bright's disease was not followed even by puffiness. In one case the patient was puffy, without any other indication of Bright's disease.

### VIII. *On a Cycle in Disease.*

In the cases given the temperature did not run an equable course, neither remaining at the same temperature throughout; but formed cycles, composed of a variable number of days, each cycle, however, being composed of the same number of days in the same patient. The cycles in the great majority of cases were composed of five days.

